# Theta-gamma coupling: nonlinearity as a universal cross-frequency coupling mechanism

**DOI:** 10.3389/fnbeh.2025.1553000

**Published:** 2025-06-23

**Authors:** Alex Sheremet, Yu Qin

**Affiliations:** ^1^Engineering School of Sustainable Infrastructure and Environment, University of Florida, Gainesville, FL, United States; ^2^Department of Neuroscience, McKnight Brain Institute, University of Florida, Gainesville, FL, United States

**Keywords:** cross frequency coupling, theta-gamma interaction, nonlinear neural activity, two-equation leaky-integrate-and-fire, gamma instability

## Abstract

The Cross Frequency Coupling (CFC) phenomenon is defined as a statistical correlation between characteristic parameters neural oscillations. This study demonstrates and analyzes the nonlinear mechanism of the CFC, with a focus on the coupling between slow and fast oscillations, as a model for theta-gamma coupling. We first discuss the usage of the spectrum/bispectrum CFC measure using experimental data. As a physical paradigm, we propose the concept of a Class II neural population at low activity: neurons fire intermittently, and the time spent in the subthreshold regime is much larger that the duration of an action potential. We verify the emergence of fast oscillations (gamma) using a direct numerical simulations (DNS) of a population of Hodgkin-Huxley neurons forced by a slow theta oscillation. To deconstruct the mechanism, we derive a mean field approximation based on a reduction of the Hodgkin-Huxley model to a two-equation leaky-integrate-and-fire (LIF) model. Under theta forcing, mean field model generates gamma oscillations; the solutions exhibit spectrum/bispectrum CFC patterns that agree qualitatively with both the DNS model and experimental data. For the theta-gamma coupling problem, the mean field model may be linearized using an asymptotic expansion. The analytical solution of the linear system describes theta-gamma interaction as a gamma stabilization/destabilization cycle and provides explicit expressions of the gamma amplitude and frequency modulation by theta. The results demonstrate that nonlinearity as a universal/unifying mechanism of all CFC types.

## 1 Introduction

The Cross Frequency Coupling (CFC) phenomenon may be defined as a statistical correlation between some characteristic parameters of two LFP “rhythms” (Jensen and Colgin, 2007; Jirsa and Müller, 2013; Hyafil et al., 2015). The coupling of theta and gamma rhythms is a well known example, identified in rat hippocampus (e.g., Soltesz and Deschênes, 1993; Bragin et al., 1995; Rosenblum et al., 2000; Colgin et al., 2009; Lisman, 2005; Belluscio et al., 2012; Colgin, 2014; Pernía-Andrade and Jonas, 2014), in the visual cortex of primates (Whittingstall and Logothetis, 2009; Mazzoni et al., 2011), and in humans (Canolty et al., 2006), but CFC phenomena also include sharp waves/ripples (Buzsáki et al., 1992; Skaggs et al., 2007; Bragin et al., 1999; Buzsáki, 2006; Clemens et al., 2011; Buzsáki and da Silva, 2012), and slow oscillations/spindles (Marshall et al., 2006; Rasch and Born, 2013; Staresina et al., 2015; Ladenbauer et al., 2017). CFC has been associated with a long list of cognitive activity aspects, such as local processing and communication between thalamus and neocortex (Fitzgerald et al., 2013); formation of short term memories (Colgin et al., 2009; Lisman and Jensen, 2013; Pernía-Andrade and Jonas, 2014; Lega et al., 2014; Bergmann and Born, 2018); information extraction for decoding motor imagery types (Gwon and Ahn, 2021); as a mechanism for the orienting response (Isler et al., 2008); and many others.

However, despite decades of research, the physical foundation of the CFC process is not well understood (e.g., Jensen and Colgin, 2007; Jirsa and Müller, 2013; Hyafil et al., 2015). The CFC classification into PP, PA, AA, PF, and AF subtypes, basically all two-letter combinations of A, F, and P (amplitude, frequency, and phase) seems taxonomic than rather founded on some mechanistic distinction. Only three (PP, PA, and AA) types have been identified experimentally (Cohen, 2008; Belluscio et al., 2012; De Hemptinne et al., 2013; Munia and Aviyente, 2019; Qin et al., 2021; Chehelcheraghi et al., 2017). Of these, only the PP type, also known as *n*:*m* phase synchronization (e.g., Ermentrout, 1981; Jirsa and Müller, 2013), has found a mechanistic description in the limit cycle oscillator model (e.g., Kuramoto, 1975, 1984; Ermentrout, 1991; Strogatz, 2000; Stiefel and Ermentrout, 2016; Nakao, 2016). The origin of other CFC subtypes remains unclear.

This situation is surprising, because all CFC subtypes are in fact aspects of a single overarching physical phenomenon, the coupling of activity across scales—if we think of frequencies as time scales. It is interesting to note that a unifying statistical CFC test does exists: the bispectrum (Jirsa and Müller, 2013; Sheremet et al., 2016, 2018b) subsumes other statistical CFC measures (Kovach et al., 2018). The bispectrum also arises naturally as a statistical characteristic of nonlinear dynamics, and as such it is a universal foundation of CFC physics (Sheremet et al., 2020, 2019). Nonlinearity is of course also the PP coupling mechanism.

Examining the “solved” PP case may shed some light. The limit cycle oscillator model is a highly simplified mathematical description of a population of independent oscillators in a state of sustained periodic oscillations. Oscillators have fixed amplitudes and frequencies, but are allowed weak interaction, which causes mutual phase shifts (Stiefel and Ermentrout, 2016). Because the physical state of the population is completely described by their phases, the model also known as a “phase equation” representation.^1^

Because it assumes that amplitudes are constant, a phase model cannot describe AA or AF coupling. To understand amplitude related CFC we need a model that accounts for significant amplitude variations. However, we cannot simply go back to the “parent” model of the phase equation and remove the constant amplitude restriction, because a “parent” model does not exist for neural populations. We need to construct it.

For this we need a physical paradigm of neural activity. We reason that understanding amplitude effects requires a model capable of describing arbitrarily low neural activity, in other words, amplitude emergence. But amplitude emergence means that 1) new frequency emerge; and 2) there is an active energy exchange between frequencies. Transfers of energy across frequencies generate *phase correlations*, in other words, CFC. These two points are in fact the very definition of nonlinearity.

**Figure 1:**
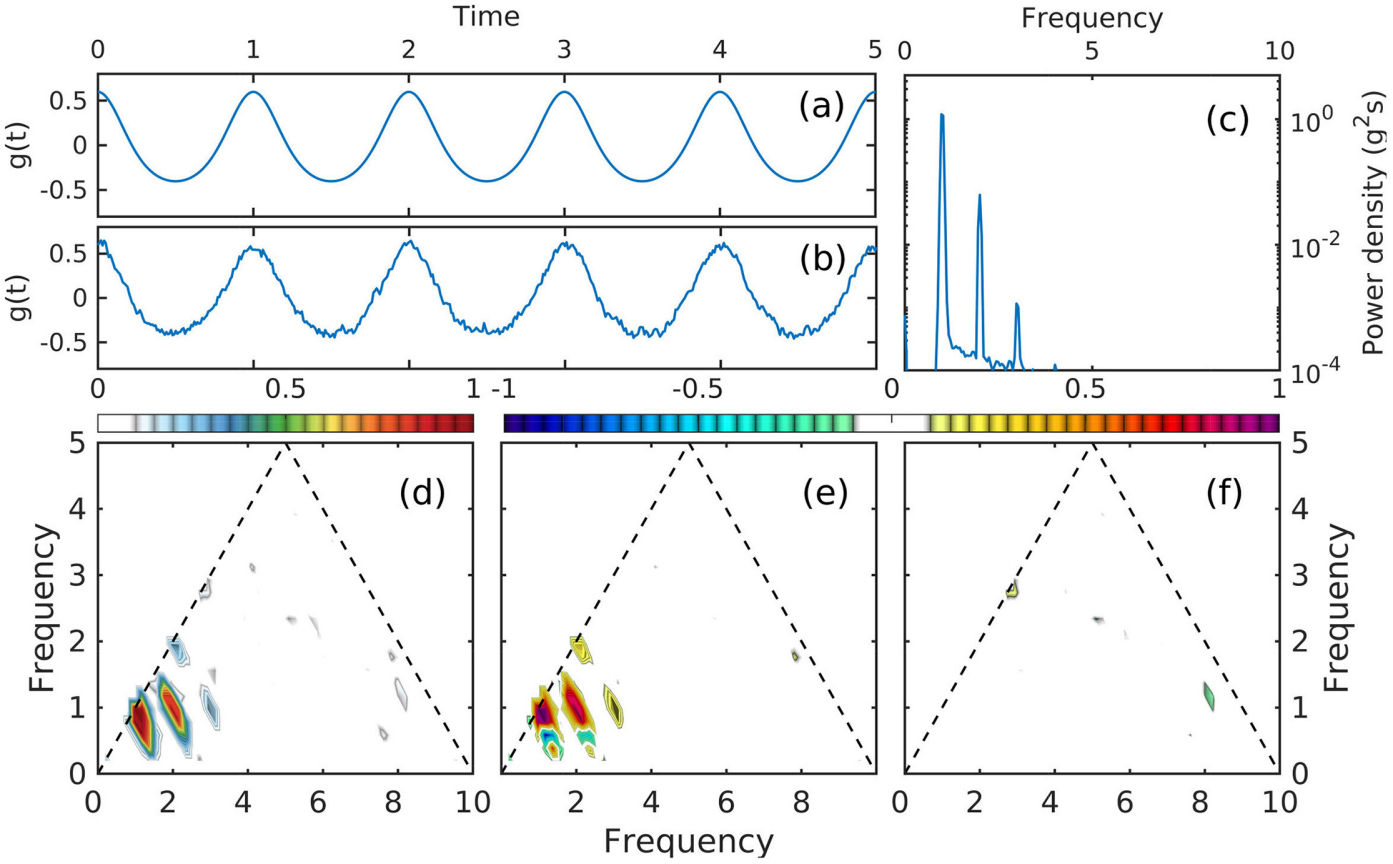
**(a)** Example of membrane potential evolution of a pyramidal neuron in CA1; 1-s segment intracellular recording data (blue; courtesy of D. English, please refer to English et al., 2014 for details). The red line represents subthreshold fluctuations, with action potentials removed using a simple thresholding algorithm applied to the voltage time derivative). Circles mark the initiation of an action potential (arbitrary voltage reference). **(b)** PDF function of the threshold for action potential (gray rectangle in **(a)**. In this time series: total time: 3,059 s; total action potentials: 326 seconds; number of action potentials: 25,907; average firing rate of 8.49 Hz.

Therefore, we focus here on neural populations at low activity. In this state, neurons fire intermittently (as opposed to periodically, as limit cycle oscillators) and spend most of the time in the subthreshold regime (e.g., *u* < −55 mV, where *u* is the membrane potential; see Figure 1). Action potentials are random and sparse, separated by time intervals at least an order of magnitude larger than the duration of an action potential, and caused by the superposition of presynaptic excitation received from the large number of neighborhood neurons. These states are relevant for populations of recurrently connected Class II neurons (e.g., Hodgkin, 1948; Ermentrout and Terman, 2010; Gerstner et al., 2014) where each neuron has of many random and weak connections (possibly thousands, e.g., Hoppensteadt and Izhikevich, 1997; Muller et al., 2018).

In these states, the population may transition from a chaotic firing to some degree of oscillatory organization into spontaneous neural assemblies. This organization is dynamically distinct from synchronization of fixed oscillatory circuitry. In this context, the concept of “amplitude” is related to collective neural activity, and is measured by the LFP spectrum. Remarkably, these states exhibit significant CFC patterns, measurable using the general bispectrum test (e.g., Sheremet et al., 2016, 2019).

The goal of this study is to demonstrate the nonlinear foundation of CFC. We use two modeling approaches: direct numerical simulations (DNS) of a population of Hodgkin-Huxley neurons (e.g., Hodgkin and Huxley, 1952), and a simplified mean field approximation based on a two-equation leaky integrate-and-fire (LIF2) model derived from the Hodgkin-Huxley model. The models represent dual, microscopic-macroscopic descriptions. We use the DNS model as a baseline for describing nonlinearity in a neural population. It is straightforward to implement, but its results are difficult to analyze and interpret. We use the mean field approximation to deconstruct the nonlinear mechanism and obtain a simple interpretation of the CFC process. Both models are applied to study the emergence of gamma-like fast oscillations under a slow theta forcing. To identify CFC, we use the spectrum/bicoherence (Jirsa and Müller, 2013; Sheremet et al., 2016, 2018b).

Some experimental evidence is briefly discussed in section (2), where we also introduce and describe briefly the use of the spectrum/bicoherence test for CFC. Section 3 formulates the framework and scope of this study. In Section 4 we discuss direct numerical simulations of a population of fully nonlinear Class II neurons (e.g., Hodgkin and Huxley, 1952; Gerstner et al., 2014) that illustrate the emergence of oscillatory behavior. In Section 5 we derive the simplified model. Slow-fast oscillation coupling in the mean field approximation is analyzed analytically and numerically in Section 6. We summarize and discuss the results in Section 7. The Appendix provides an informal short introduction to bispectral analysis.

## 2 Some experimental evidence: the spectrum/bicoherence pair

In rat hippocampus, power spectra of local field potential (LFP) recordings exhibit two well studied rhythms: theta, with frequency *f*≈8 Hz, and gamma, a weaker family of oscillations that cover wide frequency band, approximately 70 Hz. < *f* < 120. With spatial scales in the order of 10^−2^ m and frequency in the order of 100 Hz (speeds of ~ 1 m/s), gamma classifies as mesoscopic activity (Muller et al., 2018).

A useful method to detect rhythms in LFP recordings and estimate their statistical coupling uses the Fourier spectrum and bispectrum (or its modulus, the bicoherence; see Appendix for a brief account of the bispectral estimate). This method has the advantage of requiring minimal data processing, and being comparatively free of assumptions (less prone to *a priori* fallacies). The spectrum allows for identifying the dominant LFP frequencies, as peaks of power density. The bicoherence is a measure of statistical coupling across the spectrum. If the bicoherence is statistically zero at coordinates (*f*_1_, *f*_2_), the two frequencies are statistically independent; alternatively, a statistically significant (e.g., >0.15, Sheremet et al., 2016) bicoherence peak at coordinates (*f*_1_, *f*_2_) indicates CFC between frequencies *f*_1_, *f*_2_, and *f*_1_+*f*_2_.

**Figure 2:**
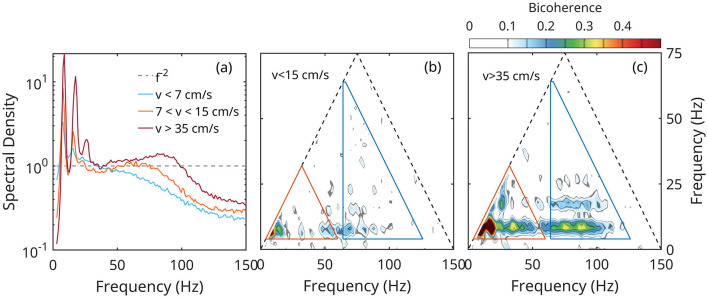
Example of CFC involving both amplitudes and phases of rat hippocampal LFP rhythms, as a function of rat running speed *v* (Sheremet et al., 2019, 2020). **(a)** LFP power density as a function of speed; the spectrum is normalized by *f*^−2^, where *f* is the frequency. **(b, c)** Bicoherence maps; peaks indicate CFC at the coordinates of the peak; triangles mark the domains of theta-theta coupling (red) and theta-gamma (blue); see text for discussion.

Figure 2 illustrates these ideas on using the transformation of LFP with the running speed (Sheremet et al., 2016, 2018a, 2020). As running speed increases, the original theta peak at *f*≈8 Hz grows and sharpens, and develops prominent secondary peaks at 16 Hz and 24 Hz, clearly visible in the spectrum corresponding to *v*>15 cm/s (red lines in Figure 2a). The spectrum also shows power accumulating in a broad gamma band spanning frequencies between approximately 70 Hz and 120 Hz (gamma oscillations). In the bicoherence map (Figures 2b, c), the growth of the theta and gamma spectral peaks is accompanied by the development of significant peaks at matching frequencies. For example, the two large peaks at coordinates (8,8) and (16,8) Hz in the bicoherence map (Figure 2c), indicate CFC between theta (8 Hz), and 16 Hz and 24 Hz spectral peaks; this identifies the 16 Hz and 24 Hz oscillations as theta harmonics. Weaker, but statistically significant bicoherence peaks match theta harmonic frequencies (domain marked red triangles in Figures 2b, c) up to the 6th, 48 Hz harmonic. This is remarkable, because there are no detectable corresponding peaks in the spectrum: the bicoherence is able to detect CFC at nascent forced frequencies with very weak amplitudes. The two wide horizontal bands parallel to the *x*-axis and extending approximately between 75 Hz and 120 Hz indicate CFC between theta and its second harmonic, on the one hand, and gamma on the other.

This brief spectrum/bicoherence analysis shows that emergence of new frequencies (theta harmonics and gamma) and their amplitude growth are intimately connected to CFC. The spectrum/bicoherence test can detect CFC at low activity levels. Below, we use this approach to test for CFC coupling.

## 3 Formulation of the problem

Here, we consider low activity levels of populations of Class II neurons. To demonstrate the nonlinear mechanism of CFC, we formulate the problem in its simplest form, retaining only the elements strictly necessary to support the emergence of new frequencies: nonlinearity, and the ability to support collective oscillations. The former is an intrinsic property of neural activity; the latter requires some negative feedback, such as inhibitory activity, adaptation, etc (e.g., Palkar et al., 2023, and references therein). For simplicity, we use here only Hodgkin-Huxley neurons (Hodgkin and Huxley, 1952); their intrinsic refractoriness will be used as negative feedback.

### 3.1 Neuron model

The Hodgkin-Huxley model (Hodgkin and Huxley, 1952; Hodgkin, 1958), describes explicitly the dynamics of ion gates. Because the model is well known, (e.g., FitzHugh, 1955; Abbott and Kepler, 1990; Kepler et al., 1992, and many others), here we only summarize its basic characteristics.

**Figure 3:**
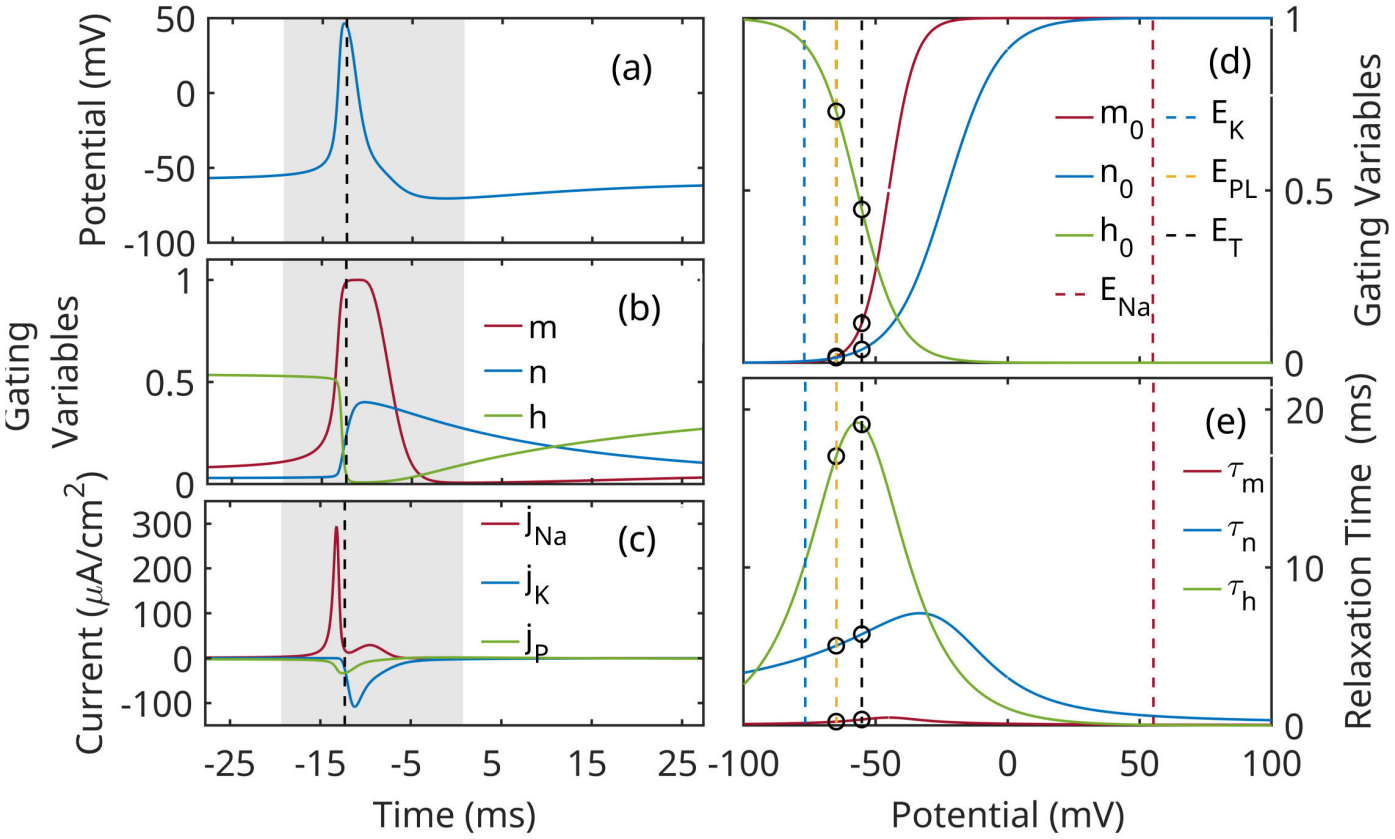
Characteristic evolution during an action potential: **(a)** membrane potential *u*; **(b)** gating variables *m*, *h*, *n*; **(c)** ion currents. The action potential event is marked by a gray rectangle. **(d)** Voltage-dependent equilibrium values of gating variables *m*, *h* and *n*; **(e)** Voltage-dependent time constants of the gating variables. vertical lines mark reversal potentials. Circles mark the limits of the subthreshold band *E*_*L*_ ≤ *u*≾*E*_*T*_, where *E*_*L*_ = −65 mV is the leaky reversal potential, and *E*_*T*_≈−55 mV is a characteristic value of the threshold potential.

The microscopic model describes the evolution of the unit surface membrane potential *u* and three gating variables *m*, *n*, and *h*


(1) $$\begin{array}{lll} \frac{du}{dt} & = & -j_{L}-j_{\text{Na}}-j_{\text{K}}+j_{Nt}, \end{array}$$

(2) $$\begin{array}{l} j_{L}=g_{L}\left(u-E_{L}\right);\;j_{\text{Na}}=g_{\text{Na}}m^{3}h\left(u-E_{\text{Na}}\right);\\ j_{\text{K}}=g_{\text{K}}n^{4}\left(u-E_{\text{K}}\right), \end{array}$$

(3) $$\begin{array}{lll} \frac{d\xi \left(u,t\right)}{dt} & = & -\kappa_{\xi }\left(u\right)\left[\xi \left(u,t\right)-\xi_{0}\left(u\right)\right],\;\xi =m,n,h, \end{array}$$

where *j*_*L*_, *j*_Na_, *j*_K_, and *j*_*Nt*_ denote ionic currents due to: sodium-potassium pump and passive transport (leaky, *L*), Na+, and K+ ion channels, and neurotransmitters, respectively. The *currents are normalized* by the capacity of the cell; *E*_*L*_, *E*_Na_, *E*_K_ are the corresponding reversal potentials; *g*_*L*_, *g*_Na_, and *g*_K_ are constant conductivity values. The evolution of gating variables *m*, *n*, and *h* (Equation 3) is described as relaxation process to equilibrium values ξ_0_(*u*) with characteristic relaxation rate κ_ξ_, or relaxation time τ_ξ_ = 1/κ_ξ_. The parameters used in the calculations below are based on Mainen et al. (1995) for a cortex excitatory neuron. The characteristic behavior of the neuron is shown in Figure 3.

### 3.2 Numerical models

The activity of a neural population activity is modeled here using direct numerical simulations (DNS) of neural populations (e.g., Kadmon and Sompolinsky, 2015; Hasegawa, 2000) and a mean field approximation (e.g., Wilson and Cowan, 1972; Jirsa and Haken, 1996, 1997; Coombes et al., 2003; Palkar et al., 2023). The former describes explicitly the dynamics each of the neurons, and can incorporate arbitrarily detailed information of microscopic brain physiology, but are numerically expensive, and produce a wealth of information that is difficult to process and interpret. The latter describe the evolution of population averages; it has direct relevance to macroscopic measurements, it is fast and relatively inexpensive numerically, but involves significant simplifications, and the relationship between its parameter space and physiological quantities is not explicit or simple to obtain.

We use direct numerical simulations of a population of Hodgkin-Huxley neurons under oscillatory forcing to demonstrate the emergence new frequencies, and to test for CFC. We use the mean field approximation to deconstruct the role of nonlinearity in the CFC generation.

### 3.3 Cautionary note about the interpretation of results

To study the nonlinear foundation of CF we leverage here three means on investigation: experimental data, and two modeling approaches, a DNS and a mean field approximation. These tools provide significantly radically different perspectives on the activity of neural populations.

The numerical models represent a highly simplified neural system, a neural mass of Class II point neurons with refractoriness, under a simple oscillatory forcing. Experimental data is infinitely more complicated that that.

The numerical models themselves are not just different, they represent two opposite perspectives: microscopic in the DNS case, macroscopic in the mean field case. In principle, they could be calibrated to produce matching results, but this is an effort beyond the scope of this study. Getting the models to agree with the experimental data is obviously impossible in the simplified setup used her.

However, our purpose is to demonstrate and investigate the nonlinear mechanism of CFC in populations of neurons at low activity levels. For this, a qualitative agreement between patterns in the CFC spectral/bicoherence test suffices.

## 4 The DNS model

The DNS model uses the Brian2 numerical simulator for spiking neural networks (Stimberg et al., 2019) to integrate equations (Equations 1–3) for a population of 1,000 interconnected neurons. Neurons receive input through 20 connections on average, and have on average 30 recurrent connections. The external forcing is modeled as Poisson time series corresponding to a firing rate of the form *F*(*t*) = *F*_0_+*A*cos2π*ft*, where *F*_0_ = 100 Hz, *A* = 10 Hz, and *f* = 8 Hz. The 8 Hz modulation emulates the theta oscillation.

**Figure 4:**
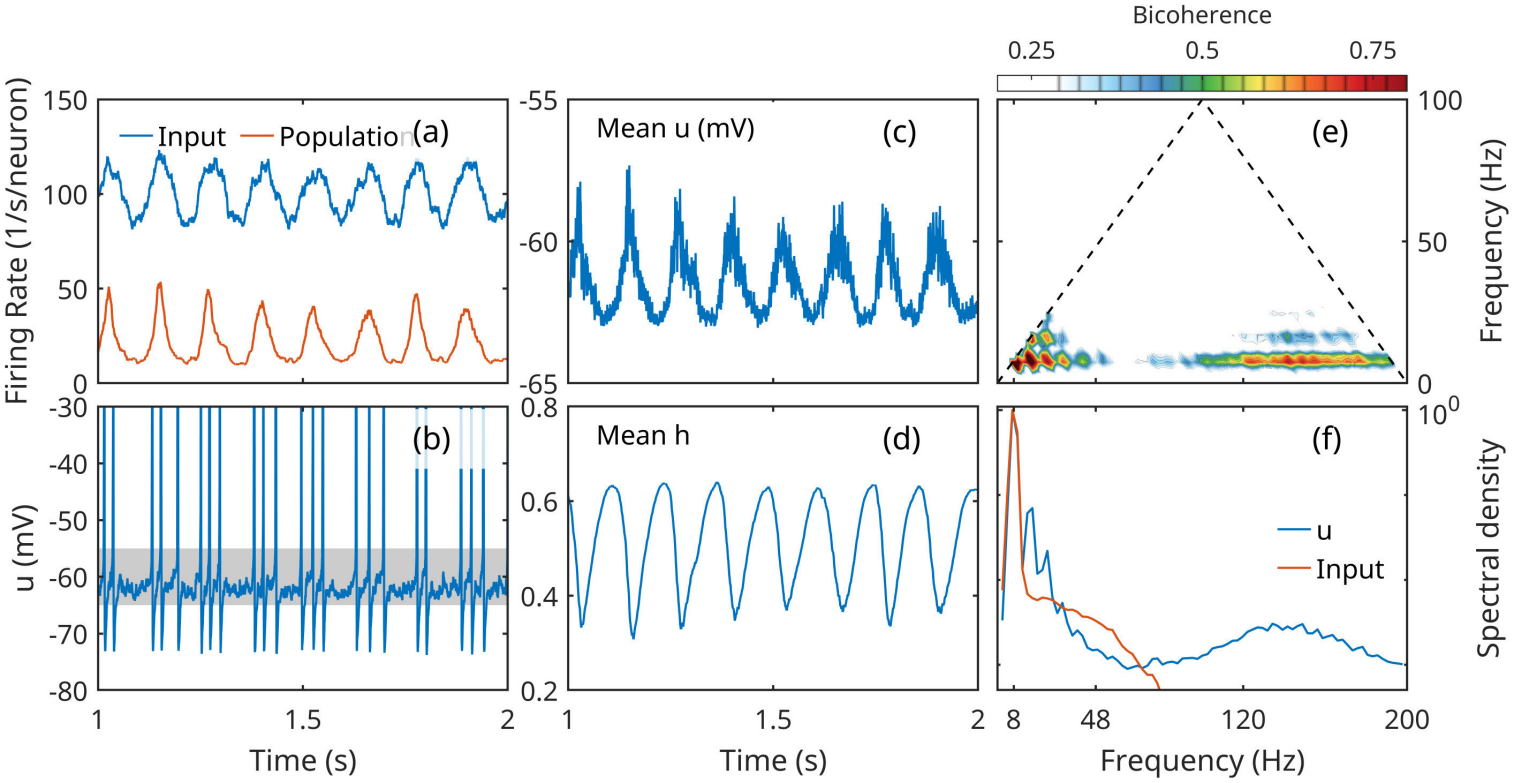
Response of a population of Hodgkin-Huxley neurons to slow oscillatory forcing. **(a)** Mean input action potential rate received by a population neuron (blue) and mean firing rate of a population neuron. **(b)** Time series of the membrane potential of neuron; the subthreshold regime is marked by a gray band. **(c, d)** Population mean membrane potential *u* and gating variable *h*. **(e)** Bicoherence of the mean membrane potential; **(f)** normalized spectral density of mean membrane potential and input firing rate.

The simulations confirm that a population of Hodgkin-Huxley neurons exhibits emergent oscillations under periodic forcing. Figure 4 summarizes some essential aspects of the population activity that illustrate our concept of low activity. The population responds to the modulated input with an oscillation on the same frequency, clearly detectable in the mean firing rate, membrane potential *u*, and gating variable *h* (Figures 4a, c, d). However, individual neurons fire intermittently (Figure 4b), with action potentials separated by large time intervals spent in subthreshold regime. The oscillations represent a spontaneous organization of collective activity oscillation, driven by input, rather than synchronization of limit cycle activity of individual neurons. (Synchronization may be achieved if the input is strong enough to drive all neurons into limit cycles).

The results also show the hallmark signs of nonlinear CFC. While the mean input is Gaussian (not skewed, and symmetric), the response of the population shows significant skewness (positive for mean *u*, negative for mean *h*), and asymmetry (negative for the mean firing rate and mean *u*; positive for the mean *h*; Figures 4a, c, d). This deformation is associated with the appearance of higher harmonics of the theta frequency. The spectral density shows four, maybe five peaks at theta harmonics, with matching peaks are prominent in the bicoherence map (Figures 4e, f).

The spectrum exhibits also a broad gamma peak approximately between 80 and 180 Hz, with the bicoherence peaks coupling gamma and theta (both 8 Hz peak and 16 Hz harmonic).

This behavior is qualitatively similar to the measurements discussed in Section 2. Compared to experimental data, the most obvious differences are the strength of the CFC effect (number and height of peaks in both spectrum and bicoherence), and the higher frequency of the “gamma” band, possibly due to the reliance of the DNS model on refractoriness as inhibitory feedback.

The results demonstrate that slow oscillatory forcing of a population of interconnected Hodgkin-Huxley neurons at low activity levels causes the emergence of new, high-frequency oscillations, that show statistically significant CFC patterns.

## 5 The mean field approximation model

To develop a mean field approximation compatible with the Hodgkin-Huxley population model used in the previous section, we first derive a lower dimension approximation of equations (Equations 1–3) and then apply a population average. The procedure follows the classical work of FitzHugh (1955), Abbott and Kepler (1990), and Kepler et al. (1992). The main distinction between their work and the present approach is the treatment of action potentials. Because we expect neurons to spend most of the time in the subthreshold regime, with intermittent and scarce action potentials (Figures 1–4b), we assume that the duration of action potentials is much smaller than subthreshold residence time scales and neglect action potentials.

### 5.1 Dimension reduction for subthreshold dynamics

The dimension reduction of equations (Equations 1–3) is based on the following observations. In the subthreshold regime *E*_*L*_ ≤ *u*≾−55 mV (see Figure 3):

(1) The small relaxation time of *m* (Figure 3e) indicates that it relaxes quickly to its equilibrium value; we neglect its explicit time dependency and replace it everywhere with *m*_0_(*u*).
(2) The dependence of *h* on *u* is weak (Figure 3d); we a write *h*(*u, t*)≈*h*(*u*_0_, *t*), with *h*_0_(*u*_0_) = *h*_00_ and κ_*h*_ = κ_*h*_(*u*_0_) = κ_*h*0_ constants *u*_0_ is a reference value, say *u*_0_ = *E*_*L*_.
(3) In the subthreshold regime *n*≪1, and it appears at fourth power in the potassium current; we neglect *n* dynamics altogether.

Therefore, the subthreshold dynamics of equations (Equations 1–3) may be approximated as


(4) $$\begin{array}{lll} \frac{d}{dt}u\left(t\right) & = & j_{L}\left(u\right)+j_{\text{Na}}\left(u,t\right)+j_{N}\left(t\right), \end{array}$$

(5) $$\begin{array}{l} \;j_{L}=g_{L}\left(u-E_{L}\right),\;j_{\text{Na}}=\frac{h(t)}{h_{00}}G(u),\text{ with}\\ G(u)=g_{\text{Na}}h_{00}m_{0}^{3}(u)\left[E_{\text{Na}}-u(t)\right], \end{array}$$

(6) $$\begin{array}{lll} \frac{d}{dt}\frac{h\left(t\right)}{h_{00}} & = & -\kappa_{h0}\left[\frac{h\left(t\right)}{h_{00}}-1\right]. \end{array}$$

This approximation is not complete, however, because is does not account for the resetting effect an action potential.

To correct this, we note that the wide distribution of the threshold membrane potential (Figure 1b; see also Gerstner et al., 2014) indicates that a fixed-value threshold criterion is a severe simplification (e.g., Koch et al., 1995; Rinzel and Ermentrout, 1998). Adopting the more natural threshold criterion *j*_Na_≈*j*_*L*_ codifies the variability of the threshold *u* value in Equation 4: the small post-spike value of *h* (Figures 3b, c) reduces the sensitivity of the sodium channel to membrane potential, and consequently, the ability of the neuron to fire.

To switch to a threshold criterion based on *j*_Na_, it is convenient to change state variable from *u* to *j*_Na_. Differentiating *j*_Na_ in Equation 5 to time, and using Equations 4, 6, after some straightforward algebra obtains


(7) $$\begin{array}{l} \frac{d}{dt}j_{\text{Na}}=\frac{h}{h_{00}}\varphi_{N}\left(j_{\text{Na}},j_{N}\right)-\kappa_{j}\left(j_{\text{Na}},h\right)j_{\text{Na}}, \end{array}$$

where φ_*N*_ represents rate of change rate *j*_Na_ induced by neurotransmitters in absence of refractoriness


(8) $$\begin{array}{l} \varphi_{N}\left(j_{\text{Na}},j_{N}\right)=hG^{'}j_{N},\;\kappa_{j}\left(j_{\text{Na}},\phi_{Nt}\right)=-\left(hG^{'}\frac{j_{L}}{j_{\text{Na}}}+hG^{'}\right)\\ \;-\frac{1}{\tau_{h0}}\left(1-\frac{h}{h_{00}}\right), \end{array}$$

and all dependencies on *u* become dependencies on *j*_Na_ and *t*. Replacing Equation 4 with 7 completes the change of variable from *u* to *j*_Na_.

We can now approximate the threshold criterion as *j*_Na_ = *j*_*T*_, where *j*_*T*_ is characteristic (constant) value, and represent the action potential resetting as a Dirac delta function (e.g., Roxin et al., 2011):


(9) $$\begin{array}{ll} \frac{d}{dt}\frac{j_{\text{Na}}}{j_{T}} & =\frac{h}{h_{00}}\varphi_{N}-\kappa_{j}\left(\frac{j_{\text{Na}}}{j_{T}},\frac{h}{h_{00}}\right)\frac{j_{\text{Na}}}{j_{T}}-\frac{j_{\text{Na}}}{j_{T}}\delta \left(\frac{j_{\text{Na}}}{j_{T}}-1\right); \end{array}$$

(10) $$\begin{array}{ll} \frac{d}{dt}\frac{h}{h_{00}} & =-\kappa_{h0}\left(\frac{h}{h_{00}}-1\right)-\frac{h}{h_{00}}\delta \left(\frac{j_{\text{Na}}}{j_{T}}-1\right), \end{array}$$

where we assumed for simplicity that, post spike, *j*_Na_ = 0 and *h* = 0.

Equations 9, 10 represent a two-equation “leaky integrate and fire” model (LIF2). The model follows the same principle as other LIF models (e.g., Lapicque, 1907; Hill, 1936; Abbott, 1999; Dong et al., 2011), with the addition of Equation 10 accounting for excitability (*h*).

### 5.2 Mean field approximation

For a neural mass with no spatial dimensions, averaging equations (Equations 4–6) over the population and assuming that *h* and *j*_Na_ are uncorrelated obtains


(11) $$\begin{array}{lll} \frac{d}{dt}J & = & H\phi -\kappa_{j}J-\langle \frac{j_{\text{Na}}}{j_{T}}\delta \left(\frac{j_{\text{Na}}}{j_{T}}-1\right)\rangle , \end{array}$$

(12) $$\begin{array}{lll} \frac{d}{dt}H & = & -\kappa_{h0}\left(H-1\right)+\langle \frac{h}{h_{00}}\delta \left(\frac{j_{\text{Na}}}{j_{T}}-1\right)\rangle , \end{array}$$

where 〈·〉 is the average operator, *J* = 〈*j*_Na_/*j*_*T*_〉, *H* = 〈*h*/*h*_00_〉, and ϕ = 〈φ_*N*_〉, with and κ_*j*_≈κ_*j*0_ constant. The δ terms are nonzero only for neurons firing in the interval [*t, t*+*dt*], i.e., they count the action potentials in the population, recorded in the unit of time, divided by the number of neurons in the population. This quantity is the firing rate *N*,


(13) $$\begin{array}{l} N=\frac{1}{n}\overset{\nu }{\underset{j=1}{\sum }}\frac{n_{s}^{j}\tau_{s}}{dt}, \end{array}$$

where ν is the number of neurons firing in [*t, t*+*dt*], $n_{s}^{j}\tau_{sj}$ approximates the time spent firing by neuron *j*, τ_*s*_ is the mean duration of a spike, and $n_{s}^{j}$ is the number of spikes. Further writing ϕ = μ(*N*+*Q*), where *Q* is the external input, described as a firing rate, and μ is a “connectivity” parameter that describes the conversion of the presynaptic firing rates *N*+*Q* into postsynaptic *j*_*N*_ currents, Equation 11 become


(14) $$\begin{array}{l} \frac{d}{dt}J=H\mu \left(N+Q\right)-\kappa_{j0}J-N, \end{array}$$

(15) $$\begin{array}{l} \frac{d}{dt}H=-\kappa_{h0}\left(H-1\right)-NH_{T}, \end{array}$$

where *H*_*T*_ = *h*_*T*_/*h*_00_ is the threshold value of *H*. The system (Equations 14, 15) is closed by prescribing the relation between the state variable *J* and the firing rate *N*, also known as the activation function. Because τ_*s*_ is assumed infinitesimal, the time interval Δ*t* → 0 can contain an arbitrary number of spikes. In general, Equation 13 implies that *N*≥0 and can be arbitrarily large at maximum activity level *J* = 1 when all neurons fire continuously. Here, we use the activation function derived by Qin and Sheremet (2022)


(16) $$\begin{array}{l} N\left(J\right)=\alpha \frac{J}{1-J}, \end{array}$$

where α is the “susceptibility” parameter, related of the intensity of endogenous membrane potential fluctuations, which may also be understood as the susceptibility of a neuron to fire. The activation function (Equation 16) is approximately linear for *J*≪1 and is unbounded as *J*↗1.

Equations 14, 16 are the mean field approximation of the LIF2 model. The state of the neural field is described by two state variables: the mean current density *J* and the excitability *H*. Both are normalized 0 ≤ *J, H* ≤ 1; where *H* = 0 after an action potential and *H* = 1 in the resting state; and *J* = 0 after an action potential and *J* = 1 at the action potential threshold.

The equations have a straightforward interpretation in terms of energy balance. The variable *J* may be interpreted as the kinetic energy density (also, intuitively, as the “temperature”, by analogy with an ideal gas, where temperature and kinetic energy density are proportional). The firing rates *N* (internal) and *Q* (external, Equation 14) represent the energy flux into the system due to internal and external action potentials; the conversion into local kinetic energy is controlled by the excitability *H* and the synaptic connectivity parameter μ. The second term in Equation 14 is the natural dissipation rate of the kinetic energy density due leaky currents and refractoriness. The third describes energy loss due to post action potential refractoriness.

The neural mass is collectively characterized by four “material” parameters. Two characterize the mean neuron – the relaxation rates κ_*h*0_ (Equation 15) and κ_*j*0_ (Equation 15). The other two characterize the inter-neuron communication—the connectivity μ (Equation 14), and susceptibility α (Equation 16).

The mean field approximation serves the purpose of this demonstration by retaining the two elements we consider essential for CFC generation: negative feedback, represented by the excitability *H*; and nonlinearity, represented by the activation function and the first term in Equation 14.

## 6 Nonlinear coupling between slow and fast oscillations

Equations 14–16 represent an approximation of the DNS model described in Section 4, and can be used to gain insight into the CFC mechanism. For this, we use a standard asymptotic approach (e.g., Bender and Orszag, 1991) that takes advantage of the different scales of the coupled oscillations. For simplicity, all material parameters are approximated as constants.

In the theta-gamma coupling, theta is a low frequency, high power oscillation, while gamma a high frequency, low power one. This relationship may be formalized by introducing a small parameter ϵ≪1 and writing


(17) $$\begin{array}{lll} J\left(t,\epsilon t\right) & = & J_{0}\left(\epsilon t\right)+\epsilon J_{1}\left(t,\epsilon t\right)+O\left(\epsilon^{2}\right), \end{array}$$

(18) $$\begin{array}{lll} H & = & H_{0}\left(\epsilon t\right)+\epsilon H_{1}\left(t,\epsilon t\right)+O\left(\epsilon^{2}\right), \end{array}$$

(19) $$\begin{array}{l} \;N=N_{0}(\epsilon t)+\epsilon N_{1}(t,\epsilon t),\text{ where }N_{0}=N(J_{0}),\text{ and}\\ N_{1}={\frac{\partial N}{\partial J}|}_{0}J_{1}, \end{array}$$

where $\frac{\partial N}{\partial J}$ is a variational derivative. From Equation 16 one obtains


(20) $$\begin{array}{l} \frac{\partial N}{\partial J}=\alpha {\left(1-J\right)}^{-2}\approx \alpha \left(1+2J+\ldots \right). \end{array}$$

Theta (the slow oscillation, subscript 0), is represented as an order 1 component that evolves on the “slow” time ϵ*t*. Gamma, (the fast oscillation, subscript 1), is an order ϵ component and assumed to evolve on two time scales: a “fast” time *t*, with a slow modulation of ϵ*t* scale induced by theta. Substituting into Equations 14–16 and separating the powers of ϵ obtains, after some straightforward algebra, two sets of equations.

### 6.1 Leading order system

Over the fast *t* time scales the slow variables satisfy the equilibrium (no explicit time dependency) equations


(21) $$\begin{array}{l} H_{0}\left(\mu N_{0}+Q\right)=\kappa_{j0}J_{0}+N_{0}, \end{array}$$

(22) $$\begin{array}{l} \kappa_{h0}\left(1-H_{0}\right)-N_{0}H^{T}=0 \end{array}$$

Equation 21 represents the balance between incoming energy flux and energy losses due to the natural relaxation rate κ_0_ and post spike resetting. Equation 22 describes the rate of growth of population refractoriness (1−*H*) as a consequence of action potentials.

From Equation 16, $J_{0}=\frac{N_{0}}{\alpha +N_{0}}$; solving Equation 22 in *H*_0_ and substituting into Equation 21 obtains for the equilibrium firing rate the cubic equation


(23) $$\begin{array}{l} \left(1-\tau_{h0}H^{T}N_{0}\right)\left(\mu N_{0}+Q\right)\left(\alpha +N_{0}\right)-\kappa_{j0}\left(\alpha +N_{0}\right)N_{0}-N_{0}=0, \end{array}$$

where τ_*h*0_ = 1/κ_*h*0_. Keeping fixed the mean neuron material parameters κ_*h*0_ and κ_*j*0_ implies that the solutions of Equation 23 for a given external forcing *Q*_0_ are uniquely defined by the network material parameters μ and α.

### 6.2 Second order system

At order ϵ, setting $\frac{\partial N}{\partial J}{|}_{0}J_{1}\approx \alpha J_{1}$ for low activity levels obtains


(24) $$\begin{array}{l} \left[I\frac{d}{dt}-\Omega \left(J_{0},H_{0}\right)\right]\left[\begin{array}{c} J_{1}\\ H_{1} \end{array}\right]=-\frac{d}{dt}\left[\begin{array}{c} J_{0}\\ H_{0} \end{array}\right]; \end{array}$$

where *I* is the the identity matrix, and Ω is a 2 × 2 matrix with entries


(25) $$\begin{array}{l} \Omega_{11}=H_{0}\mu \alpha -\kappa_{j0}-\alpha ,\;\Omega_{12}=\mu \alpha J_{0}+Q,\;\Omega_{21}=-\alpha H^{T},\text{ and}\\ \Omega_{22}=-\kappa_{h0}. \end{array}$$

Equation 24 describes the stability of the equilibrium states with respect to small perturbations (*J*_1_, *H*_1_). Seeking solutions for *J*_1_and *H*_1_∝*e*^λ*t*^ obtains the eigenvalues


(26) $$\begin{array}{l} \lambda_{1,2}=\kappa \pm i\omega ,\text{ with }\kappa =\frac{1}{2}T,\;\omega =\frac{1}{2}\sqrt{\Delta },\text{ and}\\ \;\Delta =T^{2}-4D, \end{array}$$

where *T* and *D* are the trace and the determinant of Ω,


(27) $$\begin{array}{ll} T & =\Omega_{11}+\Omega_{22}=\mu \alpha H_{0}-\kappa_{j0}-\alpha -\kappa_{h0}, \end{array}$$

(28) $$\begin{array}{l} D=\Omega_{11}\Omega_{22}-\Omega_{12}\Omega_{21}=-\kappa_{h0}\left(\mu \alpha H_{0}-\kappa_{j0}-\alpha \right)\\ \;+\alpha H^{T}\left(\mu \alpha J_{0}+Q_{0}\right). \end{array}$$

**Figure 5:**
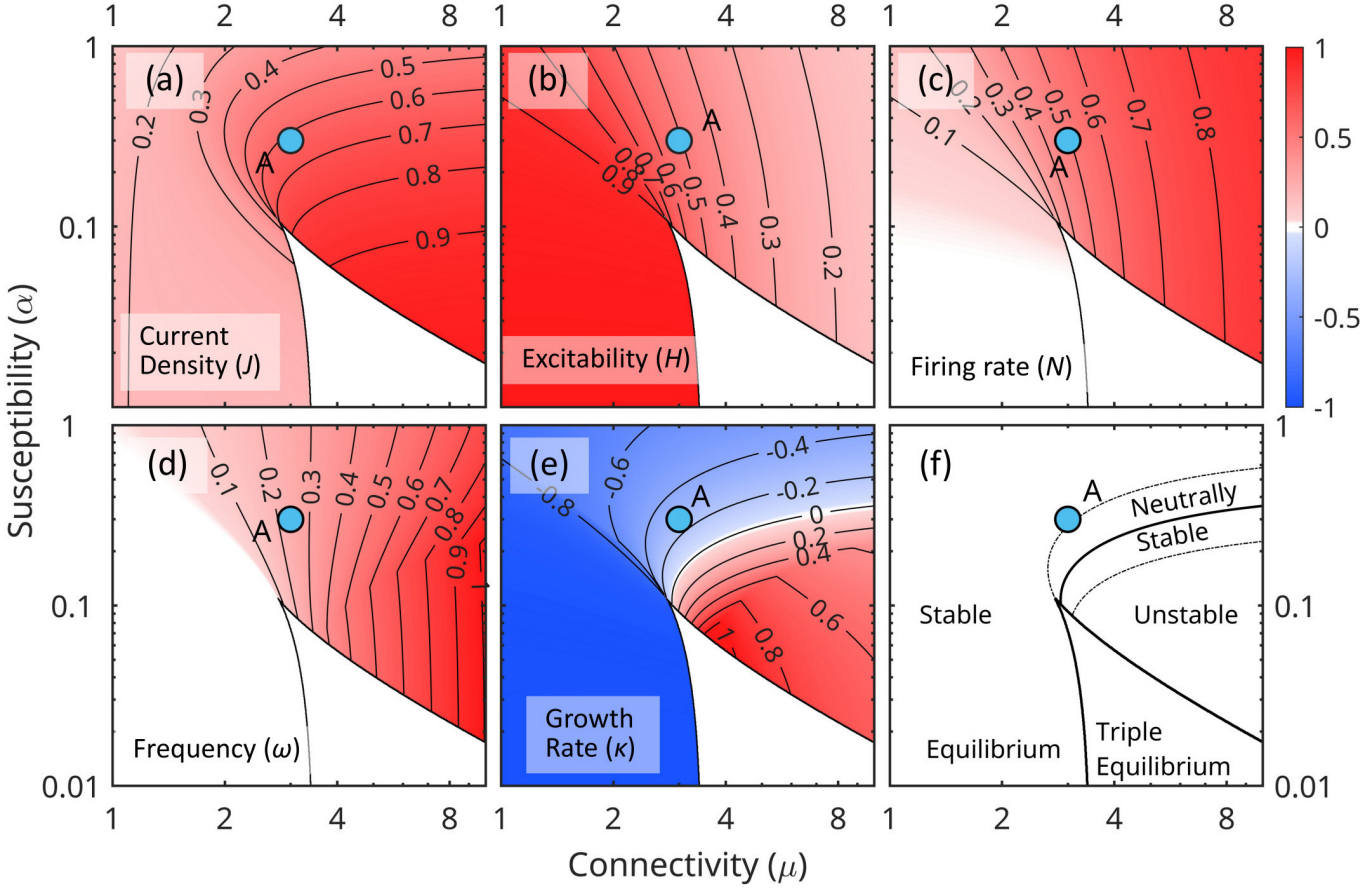
Distribution of characteristic parameters of the linearized equations (Equations 23, 24) in the (μ, α) parameter space for constant forcing *Q*_0_. Each (μ, α) point in plane represents a unique networks configuration. **(a)** Current density *J*. **(b)** Excitability *H*. **(c)** Firing rate *N*. **(d)** Frequency ω. **(e)** Growth rate κ. **(f)** Schematic map of the stability of equilibrium states. The point *A* marks the weakly stable configuration used in the numerical simulations.

The stability of equilibrium states is also completely defined by the network parameters μ and α. Equilibrium states are unstable to small perturbations if growth rate κ>0 and stable if κ ≤ 0; if Δ < 0, the perturbations are oscillatory with eigenfrequency ω. Figures 5b, c shows the stability characteristics of the equilibrium states.

### 6.3 Linear equilibrium and stability

Figure 5 shows the characteristics of the linearized mean field Equations 23, 24 in the (μ, α) parameter space.

Equilibrium states correspond to the real roots of Equation 23. Triple equilibrium states exist for low susceptibility α and strong connectivity μ (typically with two stable points separated by an unstable one) where they occupy the cusp-like white region (this is a typical feature for this kind of systems, e.g., Ermentrout, 1998). Low susceptibility maintains stability at low activity by reducing the effect of endogenous activity on firing rates; strong connectivity ϵ maintains stability at higher activity levels by sustaining a high firing rate and recapturing a large amount of the energy released.

Low connectivity μ and low susceptibility α (lower left corner) correspond to an inefficient conversion of *N*_0_ energy fluxes, and the system is driven primarily by external forcing *Q*. The opposite is true for high connectivity and susceptibility (upper right corner). As *J*_0_ and *N*_0_ increases, higher order terms in the expansion (Equations 17–19) become important, the relationship between (*J*_0_, *N*_0_) and external input *Q*_0_ weakens, and the stability of the equilibrium point decreases.

### 6.4 The nonlinear CFC mechanism for slow/fast oscillations

Because the system of equations (Equation 24) is linear, it may be solved using elementary methods. Without going into details, if ω≠0, the eigenvalues (Equation 26) are distinct and the system may be uncoupled by diagonalizing the Ω matrix


(29) $$\begin{array}{l} \left(I\frac{d}{dt}-\Lambda \right)P_{1}=-\frac{d}{dt}P_{0},\;P_{0,1}=S\left[\begin{array}{c} J_{0,1}\\ H_{0,1} \end{array}\right] \end{array}$$

where Λ is a diagonal matrix with entries λ_1, 2_ and *S* is the matrix of eigenvectors. The solutions of the problem (Equation 29) have the form


(30) $$\begin{array}{l} \;P_{1}^{j}=A^{j}(t)e^{-i\theta^{j}},\text{ with }\theta^{j}=-i\int^{t}\lambda (\zeta )d\zeta ,\text{ and}\\ A^{j}(t)=-\int^{t}\frac{dP_{0}^{j}(\zeta )}{d\zeta }e^{i\int^{\zeta }\lambda (\xi )d\xi }d\zeta . \end{array}$$

where $P_{0,1}^{j}$ are the *j*-th, (*j* = 1, 2) components of the vectors *P*_0, 1_, and λ is one of the two eigenvalues.

Note that in Equation 30 the amplitude and *A*^*j*^, the phase θ^*j*^, and frequency λ of the fast (gamma) oscillation P*j_1_ all depend on the variables *J*_0_ and *H*_0_ that characterize the slow (theta) oscillations via the eigenvalues λ_1, 2_. This solution describes explicitly the role of nonlinearity in coupling between *all theta and gamma characteristics parameters*, including all combination of amplitudes, phases, and frequencies.

The linearization achieved by expansion (Equations 17–19) preserves the nonlinear terms, but breaks them into factors of different scales and assigning their contribution accordingly. For example, the nonlinear term *HJ* in Equations 14–16 appears in Equation 24 as *H*_0_*J*_1_, mixing slow (theta, subscript 0) and fast variables. This reduces the evolution of the slow component (*J*_0_, *H*_0_) as sequence of equilibrium states (Equations 21, 22), and describes the fast oscillations (*J*_1_, *H*_1_) as a transient process of adjustment to the next equilibrium state (Equation 24).

**Figure 6:**
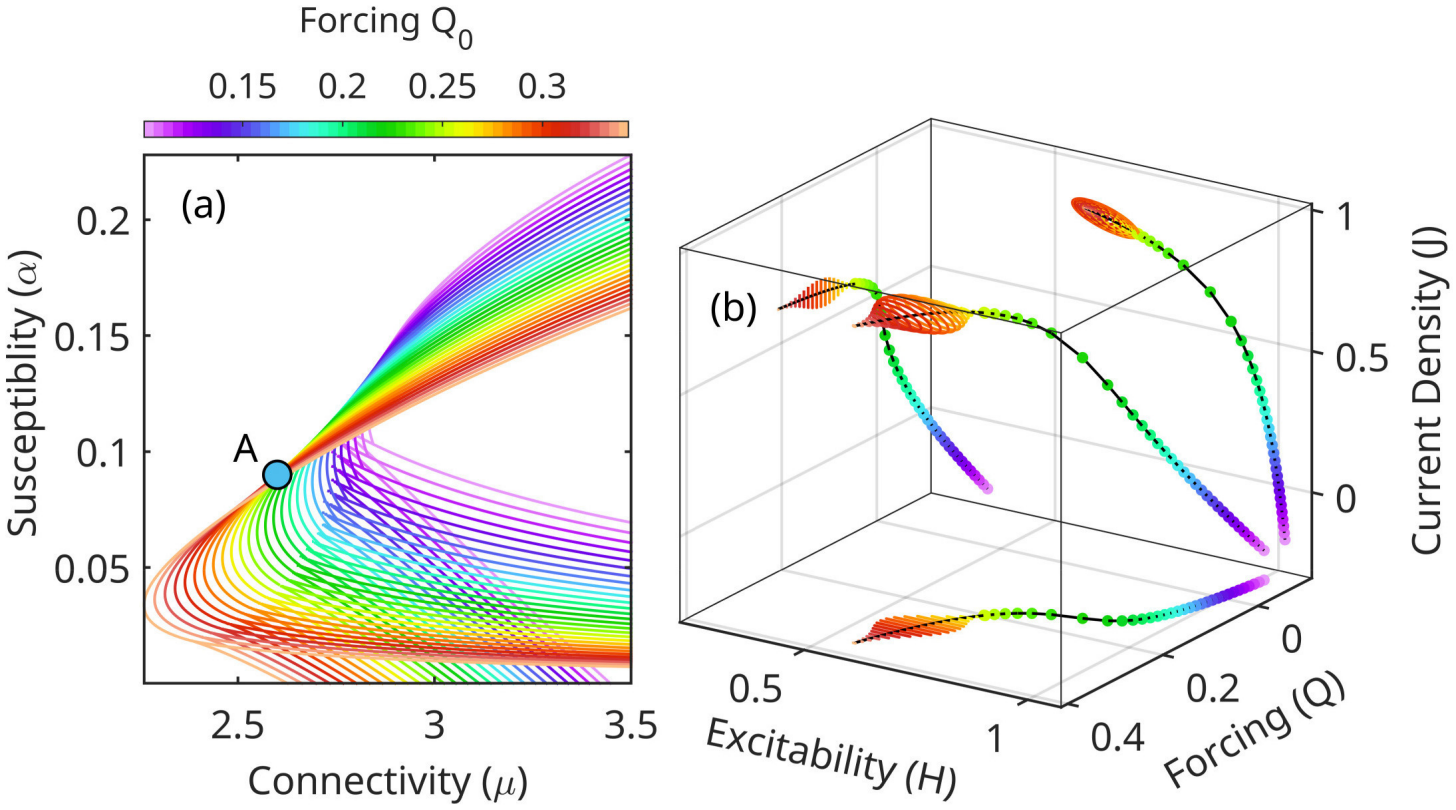
**(a)** Shifting of equilibrium stability as a function of forcing *Q*_0_ (Equation 23). Compare with Figure 5. As the forcing increases, the configuration *A* becomes unstable. In the linearized model (Equation 24) the high frequency oscillations are unbounded. **(b)** In fact, the full nonlinear system undergoes a Hopf bifurcation from node to stable orbits. Theta-gamma CFC may be visualized as a slow *Q*_0_ running through this range of values, periodically destabilizing and re-stabilizing the high frequency oscillations (gamma, limit cycles).

Because throughout this evolution the system is approximately at equilibrium, each state may be represented in the (μ_*A*_, α_*A*_), Figure 5. A slowly varying forcing *Q*_0_ shifts the topology of equilibrium regions with respect to a fixed point *A* = (μ_*A*_, α_*A*_). As *Q*_0_ increases, the triple equilibrium cusp flattens, and shifts to a lower position relative to *A* (Figure 6a). The values of current density *J*, the eigenfrequency ω and the growth rate κ at point *A* also increase. Stability decreases globally in the (μ, α) space.

In summary, the linearized equations (Equations 23, 24) describe the nonlinear mechanism of CFC between slow and fast oscillations (theta-gamma) as a cyclic destabilization of the eigenfrequencies of the system.

### 6.5 Numerical simulations

The validity of the linearized equations (Equations 23, 24) is limited by the assumptions used by expansion (Equations 17–19). Whether it is realistic of not to assumes that theta and gamma are separated in both power and frequency by, say, ϵ~10^−2^, such a scaling only describes short time behavior, because unstable eigenoscillations can grow over time and break the original ordering. To obtain long term solutions, Equations 14–16 must be integrated numerically.

#### 6.5.1 Numerical setup

**Figure 7:**
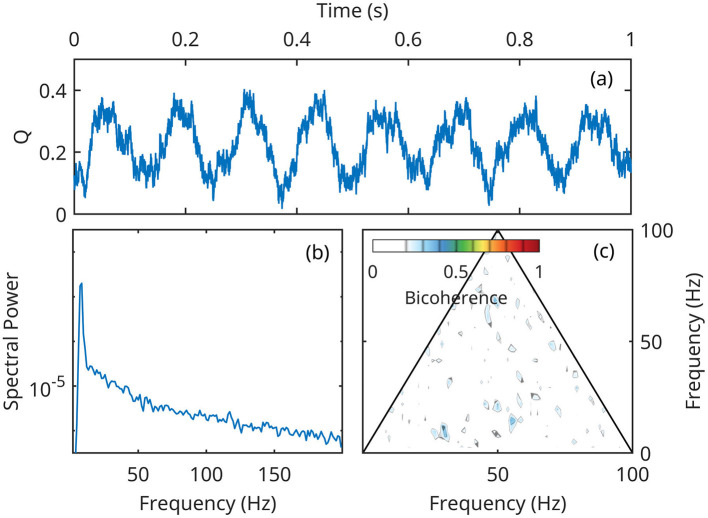
A segment of the time series *Q*(*t*) used as forcing in both linear and nonlinear numerical simulations. **(a)** A 1-second segment of the forcing time-series; **(b)** Spectral density of *Q*(*t*) power; **(c)** *Q*(*t*) bicoherence.

For numerical integration of the full system of equations (Equations 14–16), we represent forcing *Q* as a non-skewed, symmetric stochastic process with narrow-spectrum Gaussian pink noise ∝*f*^−1.5^ superposed on a monochromatic oscillation of 8 Hz (Figure 7). The noise is added in order to provide a frequency smoothing for spectral estimators, but has otherwise no dynamical effect. By construction, the forcing time series does not contain any cross-frequency coupling, therefore its spectrum only exhibits the 8 Hz peak and the bicoherence is statistically zero (Figures 7b, c).

Here, we present numerical solutions for point *A* in Figures 5, 6, chosen because it is weakly stable at approximately middle of the *Q* oscillation range (Figure 6a; see also Figure 5e). In these conditions, eigenoscillations are stable throughout most of the lower forcing values, but unstable at high *Q* values.

To enable some comparison with measurements, we construct here a mock LFP by the simple superposition


(31) $$\begin{array}{l} \text{LFP}\sim \left(Q+J\right). \end{array}$$

In the CA1 region in the hippocampus, for example, *Q* might represent external synaptic excitation excitation though Schaffer collateral path, while *J* might represent the current source near *stratum pyramidale*. Both these signals contribute to LFP recordings in the rat hippocampus. However, this description does not account for other synaptic and somatic activities such as spiking and hyperpolarization activities, and other ionic processes (Buzsáki et al., 2012).

#### 6.5.2 Results

**Figure 8:**
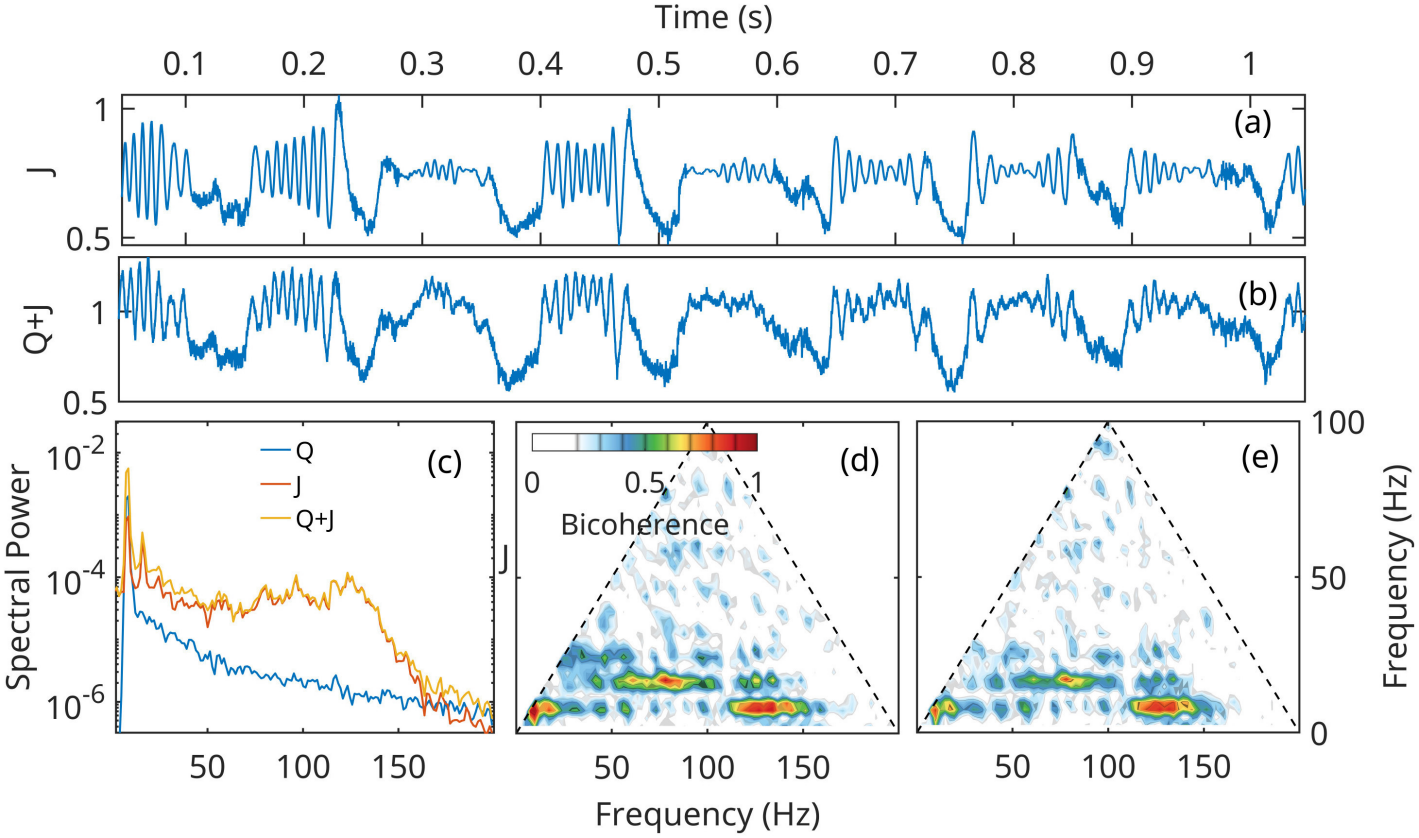
Numerical solution of the linear approximation (Equation 24) for neural network *A* (Figure 6), under the forcing *Q* defined in Figure 7. **(a, b)** time current density and mock LFP. **(c)** Spectral densities of current density *J*, forcing *Q* and *Q*+*J*, the mock LFP. **(d, e)** Bicoherence of the current density *J*, and *Q*+*J*, mock LFP.

**Figure 9:**
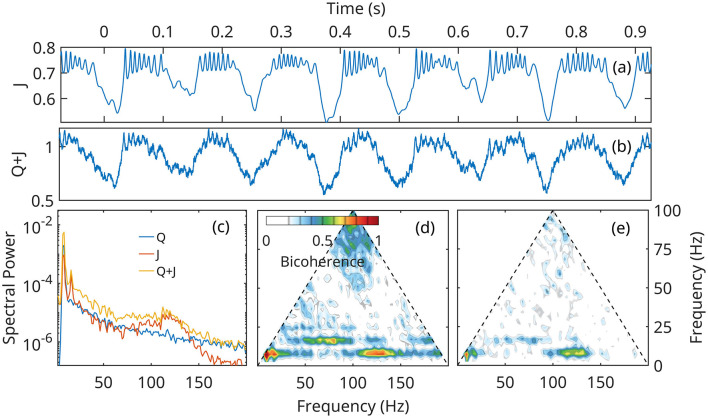
Numerical solution of the fully nonlinear mean field approximation (Equation 14–16) for neural network *A* (Figure 6), under the forcing *Q* defined in Figure 7. **(a, b)** time current density and mock LFP. **(c)** Spectral densities of current density *J*, forcing *Q* and *Q*+*J*, the mock LFP. **(d, e)** Bicoherence of the current density *J*, and *Q*+*J*, mock LFP.

Figures 8, 9 show that numerical solutions of the linearized (Equation 24) and the fully nonlinear mean field approximation (Equation 14–16) are qualitatively similar behavior. The gamma stabilization/destabilization cycle is obvious in the time series of the current density (Figures 8a, 9a). The effect is dampened in the mock LFP by the contribution of the *Q* (Figure 7a).

However, In the linearized representation, fast gamma oscillations are described as noise preferentially amplified at unstable eigenfrequencies, and can grow to arbitrarily large values. In the fully nonlinear model, the growth mechanism is initially the same, but as the gamma amplitudes increase, nonlinear terms become important and limit the growth. The solutions of the nonlinear system remain globally stable, and exhibit a bifurcation of the supercritical Hopf type (Figure 6b). This effect limits the power in the gamma band, as seen by comparing spectral densities (Figures 8c, 9c).

Theta harmonics are visible in both the spectrum and bicoherence. The bicoherence also exhibits peaks indicating significant phase correlations between theta and gamma (Figures 8c–e, 9c–e). The patterns of CFC between theta and gamma are in qualitative agreement with DNS results and experimental data. The also confirm that CFC between theta and gamma can arise strictly as a nonlinear response of the neural network to a Gaussian forcing.

## 7 Summary and discussion

The goal of this study is to demonstrate and analyze the nonlinear foundation of the CFC phenomenon.

The modeling framework is based here on a paradigm of “irregular”, low-intensity neural activity, defined as a state where neurons fire intermittently and spend most of the time in the subthreshold regime. These conditions allow neural population to organize into oscillatory patterns, with amplitudes measured by the LFP spectrum. These states are relevant for populations of Class II neurons, and have been shown to exhibit significant CFC bispectral patterns (e.g., Sheremet et al., 2016, 2019).

The nonlinear low-activity paradigm is supported by the analysis of LFP rat hippocampus recordings. The LFP spectrum and bicoherence (Jirsa and Müller, 2013; Sheremet et al., 2016, 2018b) shows distinctive CFC patterns that indicate coupling between theta and its harmonics, and between theta and gamma. Importantly, the growth of theta-gamma coupling with the power (amplitude) of the oscillations indicate that amplitude and phase types of CFC are related.

The numerical modeling framework discussed here has two components: a DNS model of interacting (Hodgkin and Huxley, 1952) neurons, and a simplified mean field approximation based on two-equation LIF model. The mean field equations are nonlinear, and retain the negative firing rate feedback, but have a much simpler structure than the DNS population model. In both models, the neural population is forced by a slow (theta) oscillation. In both models, Spectral/bicoherence of the mean membrane potential time series exhibit patterns similar to LFP recordings.

The DNS model is used to verify the nonlinear CFC mechanism directly. However, because the complexity of the DNS model makes further analysis difficult, to understand nonlinear CFC mechanism we turn to the mean field approximation model. An asymptotic expansion that takes advantage of the scale differences between theta and gamma can readily be used to linearize the system. This provides a few notable results:

(1) The linearized model describes the coupling between theta and gamma as a eigenfrequency (gamma) destabilization/restabilization cycle forced by theta: underlying mechanism of gamma forcing by theta is nonlinearity.
(2) The analytical solution of the linearized equations gives an explicit expression of the effect of nonlinearity on all gamma characteristics: amplitude, phase, and frequency. This demonstrates the role of nonlinearity as a unifying mechanism for all CFC types identified in literature. Indeed, the simplified modeling framework we use here allows for writing explicitly (Equations 26, 30) the relationship between all characteristics of the slow and fast (theta-gamma) oscillations: amplitude, phase, and frequency.
(3) The “gamma” frequencies produced by our simulations are different (higher) than experimentally observed. This is likely the result of relying on the short-time intrinsic Hodgkin-Huxley refractorinessU for negative feedback. Indeed, from Equations 24–30, the frequency λ (Equation 26) of the fast oscillation depends on the characteristics decay rates κ (Equations 27–28), originally defined in Equation 6 for the refractoriness, and Equation 8 for the sodium current. Note that in this mechanism, gamma oscillations are indeed controlled by the time scale of the negative feedback (e.g., in Equation 15, τ_*h*0_ → 0 implies *H* → 1 and nonlinearity is disabled). However, further research into this dependency is beyond the scope of this study, and would be of doubtful usefulness, given the simplicity of the models used here (inhibition largely overrides these effects).

These results show that, under theta forcing, a population of Hodgkin and Huxley (1952) neurons can spontaneously generate high frequency (gamma) oscillations due to the intrinsic nonlinearity of the neural activity. The theta-gamma system of oscillations has CFC characteristics similar to observations. This suggests the nonlinearity of brain activity is a fundamental mechanism for the generation of theta-gamma CFC (see also Sheremet et al. 2019, 2020). This is consistent with general physics results and theories for the evolution of large systems as diverse as water waves, plasma, aggregation-fragmentation in particulate systems, chemical reactions, etc (e.g., L‘vov 1998; Zakharov et al. 1992; Connaughton et al. 2006; Proment et al. 2009). Our results suggest that nonlinearity also plays a role in brain activity CFC processes and cognitive activities.

However, we should stress that albeit fundamental, nonlinearity is only one of a number of CFC mechanisms identified in brain activity at various scales, that include, e.g., the celebrated ING/PING mechanisms based on inhibition, as well as interaction between different brain regions (e.g., Bartos et al., 2007; Tiesinga and Sejnowski, 2009; Buzsáki and Wang, 2012, and many others). Further research is needed to understand the importance of the nonlinear element in this complex context of brain activity.

## Data Availability

The original contributions presented in the study are included in the article/Supplementary material, further inquiries can be directed to the corresponding author.

## Footnotes

^1^To use a phase model one has to find oscillators. Initially, the role of oscillators was played by Hodgkin-Huxley neurons (Hodgkin and Huxley, 1952) and derivative models (e.g., FitzHugh, 1955; Abbott and Kepler, 1990; Ermentrout and Kopell, 1986, and others). In time, neural “circuitry” has also been considered, such as central pattern generators (e.g., Marder and Bucher, 2001, probably not applicable to the cortex); interacting brain regions (e.g., Chehelcheraghi et al., 2017); and others.
