# Supplementary material for: Theta-gamma coupling: nonlinearity as a universal cross-frequency coupling mechanism
Source: Front Behav Neurosci. 2025 Jun 23;19:1553000. doi: 10.3389/fnbeh.2025.1553000 (PMC12230018; doi:10.3389/fnbeh.2025.1553000)
Supplement: Supplementary file 1 [file Data_Sheet_1.pdf]

THETA-GAMMA COUPLING: NONLINEARITY AS A UNIVERSAL CROSS-FREQUENCY COUPLING MECHANISM SUPPLEMENTARY MATERIAL

Authors: A. Sheremet[1] and Y. Qin[2]

Affiliations: 1. Engineering School of Sustainable Infrastructure and Environment, University of Florida, Gainesville, FL. 32611.
2. McKnight Brain Institute, Department of Neuroscience, University of Florida, Gainesville, FL. 32610.

Correspondence: Alex Sheremet, email: alex.sheremet@essie.ufl.edu

Competing Interests: The authors declare that they have no competing interests.

Data Availability: No laboratory data were used in the research presented in this manuscript.

Funding information: This work was supported by the McKnight Brain Research Foundation, National Institute on Aging, grant number AG055544, and National Institute of Mental Health, grant number MH109548.

Key words: cross frequency coupling; theta-gamma interaction; nonlinear neural activity; two-equation leaky integrate and fire; gamma instability.

1. The bispectrum

Stochastic Fourier analysis. If $p(t)$ is a stochastic process stationary in the relevant statistics sampled at $t_j =$, the discrete Fourier direct and inverse transform is defined as [e.g., Weaver, 1989]

$$P_n = \sum_{j=0}^{N-1} p_j \exp\left(-2\pi f_n t_j\right); \quad p_j = \frac{1}{N}\sum_{n=0}^{N-1} P_n \exp\left(2\pi f_n t_j\right); \tag{1}$$

where the time and frequency grids are $t_j = j\Delta t$ and $f_n = n\Delta f$, with $\Delta f = \frac{1}{N\Delta t}$, $j,n = 0,2,\ldots,N-1$, and N is the number of points in the time domain.

The discrete cross-spectrum and cross-bispectrum estimators for three stochastic processes p, q, and r, are

$$S_n^{pq} = S^{pq}(f_n) = \langle P_n Q_n^* \rangle, \tag{2a}$$

$$B_{mn}^{pqr} = B^{pqr}(f_m, f_n) = \left\langle P_m Q_n R_{m+n}^* \right\rangle. \tag{2b}$$

where P, Q, and R denote the DFTs, angular brackets represent the ensemble average, and the asterisk denotes complex conjugation. The cross-spectra describe the frequency distribution of the variance of processes p and q, i.e., a complete characterization of the average linear structure of the Fourier representation. For two distinct processes p and q, the cross-spectrum provides a measure of their correlation, typically represented as the coherence and phase lag

$$C_n^{pq} = \frac{S_n^{pq}}{\sqrt{S_n^{pp} S_n^{qq}}}, \text{ and } \Theta_n^{pq} = \arg S_n^{pq}. \tag{3}$$

For a single stochastic process the cross-bispectrum is generally referred to as bispectrum, $B_{mn}^{ppp} \equiv B_{mn}^{p}$ (the superscript may be dropped if there is no ambiguity). A variance factor is typically used

to eliminate the variance bias in the magnitude of the bispectrum: e.g., the normalization

$$b^p_{mn} = \frac{B^p_{n,m}}{\left(\left\langle |P_n P_m|^2 \right\rangle \left\langle |P_{n+m}|^2 \right\rangle\right)^{1/2}}. \tag{4}$$

insures that $\left|b^p_{mn}\right| \leq 1$ [Haubrich and MacKenzie, 1965, Elgar and Guza, 1985]. The bispectrum arises naturally as a measure phase coupling between Fourier components of time series p: for a Gaussian time series, $\left|b^p_{mn}\right|$is statistically zero, while $\Re\left\{b^p_{mn}\right\}$ $\Im\left\{b^p_{mn}\right\}$ are related to the skewness and asymmetry of p_j [Haubrich and MacKenzie, 1965, Masuda and Kuo, 1981]. The interpretation of the bispectral components is illustrated in figure 1. he use of cross-bispectra was first proposed to describe the third order statistics of ocean waves ([Hasselmann et al., 1963]. Bispectral analysis has a wide application in nonlinear systems, ranging from water waves, large scale interplanetary scintillation, plasma turbulence, to small scale pitch detection, image reconstruction, and machine fault diagnosis (see, e.g., Elgar and Guza, 1985, Spicher et al., 2015, Itoh et al., 2017, Chiang and Nikias, 1990. Applied to EEG recordings [e.g., Kleiner et al., 1969, Isler et al., 2008], bispectral analysis has been used to connect cross-frequency coupling to physiological states [Bullock et al., 1997, Andrzejak et al., 2001]. and to investigate the evolution of the nonlinear character of the hippocampal LFP as a function of rat running speed. Sheremet et al. [2016]. A discussion of the bispectral estimator in relation with the nonlinear coupling estimators popular in neuroscience can be found in Kovach et al. [2018].

References

R. G. Andrzejak, K. Lehnertz, F. Mormann, C. Rieke, P. David, and C. E. Elger. Indications of nonlinear deterministic and finite-dimensional structures in time series of brain electrical activity: Dependence on recording region and brain state. *Physical Review E*, 64(6):061907, 2001.

T. H. Bullock, J. Z. Achimowicz, R. B. Duckrow, S. S. Spencer, and V. J. Iragui-Madoz. Bicoherence of intracranial eeg in sleep, wakefulness and seizures. *Electroencephalography and Clinical Neurophysiology*, 103(6):661–78, 1997.

H.-H. Chiang and C. L Nikias. Adaptive deconvolution and identification of nonminimum phase fir systems based on cumulants. *IEEE Transactions on Automatic Control*, 35(1):36–47, 1990.

S. Elgar and R. T. Guza. Observations of bispectra of shoaling surface infragravity waves. *Journal of Fluid Mechanics*, 161:425–448, 1985.

K. Hasselmann, W. Munk, and G. McDonald. *Times Series Analysis*, chapter Bispectra of ocean waves, pages 125–139. John Wiley, New York, 1963.

R. A. Haubrich and G. S. MacKenzie. Earth noise, 5 to 500 millicycles per second: 2. Reaction of the Earth to oceans and atmosphere. *Journal of Geophysical Research*, 70(6):1429–1440, 1965.

J. R. Isler, P. G. Grieve, D. Czernochowski, R. I. Stark, and D. Friedman. Cross-frequency phase coupling of brain rhythms during the orienting response. *Brain Research*, 1232:163–172, 2008. doi: 10.1016/j.brainres.2008.07.030.

S.-I. Itoh, K. Itoh, Y. Nagashima, and Y. Kosuga. On the application of cross bispectrum and cross bicoherence. *Plasma and Fusion Research*, 12:1101003–1101003, 2017.

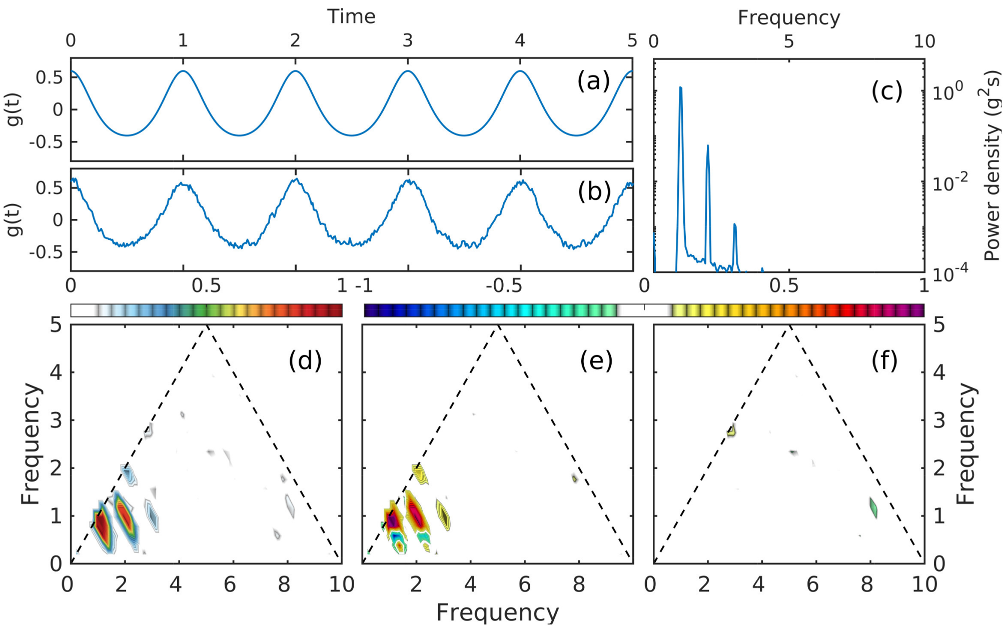

FIGURE 1. Schematic of the interpretation of the bispectrum. a) Due to the symmetries of the bispectrum [e.g., Sheremet et al., 2020] the principal (non-redundant) domain of b^p_{mn} is octant 1 in the (f,f) plane (shown here bounded by f_N (for plotting convenience, the frequency axes are not shown equal). A region in the plane (f,f) such that $b^p_{mn} > 0$ (circle) indicates cross-frequency coupling between the frequencies corresponding to the orthogonal projections of the region on the two axes (blue, centered at f_m and f_n), and the projection of the region on the horizontal axis along the second diagonal (red, centered at $f_m + f_n$). b) Smooth time series, positively skewed and symmetric. c) "Noisified" time series: weak Gaussian pink noise was added to the time series in (b), to enhance the bispectral peaks. d) Spectrum of time series (c). e-f) Components of the bispectrum of time series (c): modulus $\left|b^p_{mn}\right|$, real part $\Re\left\{b^p_{mn}\right\}$ (measure of skewness) and imaginary part $\Im\left\{b^p_{mn}\right\}$ (measure of asymmetry) bispectrum . The peaks in $\left|b^p_{mn}\right|$ indicate cross-frequency coupling between the fundamental (unit frequency) and its harmonics; they are prominent and positive in $\Re\left\{b^p_{mn}\right\}$, indicating that the coupling is associated with positive skewness; $\Im\left\{b^p_{mn}\right\}$ is statistically zero, indicating that the time series is symmetric.

B. Kleiner, P. J. Huber, and G. Dumermuth. Analysis of the interelations between frequency bands of the EEG by means of the bispectrum. *Electroencephalogr Clin Neurophysiol*, 27(7):693–4, 1969.

K.C. Kovach, H. Oya, and H. Kawasaki. The bispectrum and its relationship to phase-amplitude coupling. *NeuroImage*, 173:518–539, 2018.

A. Masuda and Y.-Y. Kuo. A note on the imaginary part of bispectra. *Deep Sea Research*, 28(3): 213–222, 1981.

A. Sheremet, S. N. Burke, and A. P. Maurer. Movement Enhances the Nonlinearity of Hippocampal Theta. *Journal Neuroscience*, 36(15):4218–30, 2016. doi: 10.1523/JNEUROSCI.3564-15.2016.

A. Sheremet, Y. Zhou, Y. Qin, J. P. Kennedy, S. D. Lovett, and A. P. Maurer. An investigation into the nonlinear coupling between CA1 layers and the dentate gyrus. *Behavioral Neuroscience*, 134(6):491–515, 2020. doi: 10.1037/bne0000366.

A Spicher, WJ Miloch, LBN Clausen, and JI Moen. Plasma turbulence and coherent structures in the polar cap observed by the ICI-2 sounding rocket. *Journal of Geophysical Research: Space Physics*, 120(12):10,959–10,978, 2015.

H.J. Weaver. *Theory of Discrete and Continuous Fourier Analysis*. Wiley Interscience, John Wiley and Sons, 1989.
